# Alkali-Activated Dredged-Sediment-Based Fluidized Solidified Soil: Early-Age Engineering Performance and Microstructural Mechanisms

**DOI:** 10.3390/ma18143408

**Published:** 2025-07-21

**Authors:** Qunchao Ma, Kangyu Wang, Qiang Li, Yuting Zhang

**Affiliations:** 1 School of Civil Engineering, Zhejiang University of Technology, Hangzhou 310015, China; 221123060117@zjut.edu.cn; 2 School of Civil Engineering and Architecture, Wuhan University of Technology, Wuhan 430070, China; 3 Power China Huadong Engineering (Shenzhen) Corporation Limited, Shenzhen 518100, China; qiangli1991@outlook.com; 4 Discipline of Civil, Surveying and Environmental Engineering, Priority Research Centre for Geotechnical Science and Engineering, The University of Newcastle, Callaghan, NSW 2308, Australia

**Keywords:** alkali-activated, dredged-sediment-based fluidized solidified soil, flowability, early-age strength, microstructural mechanisms

## Abstract

Fluidized solidified soil (FSS) has emerged as a promising material for marine pile scour remediation, yet its limited construction window and vulnerability to hydraulic erosion before sufficient curing constrain its broader application. This study systematically evaluates FSS formulations based on dredged sediment, cement partially replaced by silica fume (i.e., 0%, 4%, 8%, and 12%), and quicklime activation under three water–solid ratios (WSR, i.e., 0.525, 0.55, and 0.575). Experimental assessments included flowability tests, unconfined compressive strength, direct shear tests, and microstructural analysis via XRD and SEM. The results indicate that SF substitution significantly mitigates flowability loss during the 90–120 min interval, thereby extending the operational period. Moreover, the greatest enhancement in mechanical performance was achieved at an 8% SF replacement: at WSR = 0.55, the 3-day UCS increased by 22.78%, while the 7-day cohesion and internal friction angle rose by 13.97% and 2.59%, respectively. Microscopic analyses also confirmed that SF’s pozzolanic reaction generated additional C-S-H gel. However, the SF substitution exhibits a pronounced threshold effect, with levels above 8% introducing unreacted particles that disrupt the cementitious network. These results underscore the critical balance between flowability and early-age strength for stable marine pile scour repair, with WSR = 0.525 and 8% SF substitution identified as the optimal mix.

## 1. Introduction

Rapid industrialization and intensified water environment remediation efforts have driven the global annual production of dredged sediment to exceed one billion cubic meters. Current management practices involve disposal of over 50% of this material through landfilling or temporary stockpiling approaches that not only consume substantial land resources but also pose environmental risks due to the material’s elevated moisture content and organic load, including potential pollution and eutrophication [1,2]. Hydraulic dredging operations typically yield sediments characterized by excessive moisture content, high compressibility, and inadequate bearing capacity, necessitating pretreatment before potential engineering applications [3,4]. While conventional confined disposal facilities can eventually create usable land through self-weight consolidation and evaporation over 5–10 years, this method immobilizes land resources for extended periods while substantially elevating project expenditure [5]. Growing environmental regulatory constraints on marine disposal and unregulated storage practices further emphasize the critical demand for developing sustainable, resource-oriented utilization approaches for dredged sediments.

Existing valorization strategies for dredged sediment primarily encompass three technical routes: the production of solidified backfill materials [6,7], the development of soil amendments [8,9], and the fabrication of construction material alternatives [10,11]. Within this framework, solidification technology has gained prominence owing to its extensive engineering adaptability and economic viability. As the principal binding mechanism in conventional systems, cement-based stabilization demonstrates substantial performance enhancements. Research has confirmed that cement addition significantly improves mechanical characteristics (including unconfined compressive strength (UCS) and shear resistance) through the hydration-induced formation of C-S-H gels and crystalline phases, which densify the matrix and substantially decrease porosity [12,13,14]. Donrak et al. [15] and Liu et al. [16] further validated cement’s effectiveness in enhancing soil durability, providing solutions for construction in cryogenic environments and under elevated groundwater conditions. Nevertheless, the cement industry’s inherent limitations—characterized by intensive energy demands, dependence on finite resources, and significant CO2 emissions—have spurred research into partial substitution with industrial byproducts such as phosphogypsum (PG) and fly ash (FA). Experimental evidence has revealed nuanced substitution effects: Wan et al. [17] documented a 100% increase in 7-day UCS using a 7.5:2.5 Bayer red mud-PG blend compared with pure cement. Under 0.5% NaOH activation, Liu et al. [18] observed gradual strength development in FA-modified systems, with the C11.25F3.75 formulation showing merely 13.68% strength gain at 14 days, followed by strength reduction at higher FA contents. In advanced ternary cement–FA–GGBS systems, Liu et al. [19] achieved simultaneous rapid dewatering and strength enhancement, albeit noting shear strength deterioration beyond 15% FA incorporation. These findings collectively indicate that while industrial byproduct substitution can optimize stabilization performance, precise dosage control remains critical to preventing threshold-induced mechanical property degradation.

In contemporary engineering practice, fluidized solidified soil (FSS) has become a preferred technology for fill and remediation thanks to its pumpability, tunable low-strength behavior, and environmental benefits [20]. In underwater marine pile scour repair, however, pumped placement faces two inter-related challenges: a limited construction window due to time-dependent flowability loss and the risk of hydraulic erosion before sufficient curing, which can cause premature cracking and compromise structural integrity. Sustaining extended flowability remains essential to enabling dense scour pit filling through self-leveling behavior; simultaneously, mitigating early hydraulic erosion is particularly crucial for low-strength FSS to preserve integrity before full strength development. However, research has predominantly addressed either initial pumpability or long-term durability, leaving significant knowledge gaps regarding time-dependent flowability evolution and erosion resistance mechanisms during the early-age curing period. Effective scour remediation thus demands formulations that synergistically maintain workable rheology while accelerating early strength gain—a dual-performance optimization challenge necessitating systematic investigation of early-age rheological–mechanical evolution in FSS as a prerequisite for advancing marine infrastructure rehabilitation technologies.

Silica fume (SF), a byproduct of silicon–metal smelting processes, has experienced exponential growth in global production volumes. The improper disposal of this nanoscale particulate material presents substantial environmental risks, driving an urgent need for its valorization in innovative construction materials. Characterized by an ultra-high specific surface area and exceptional pozzolanic reactivity, SF demonstrates a remarkable capacity to enhance early-age strength development through improved thixotropic regulation in cementitious matrices [21,22]. Experimental evidence has illustrated this enhancement mechanism: Luo et al. [23] achieved a 67.1% enhancement in 3-day UCS through SF incorporation in concrete systems compared with reference samples. He et al. [24] further demonstrated that 5% cement substitution with SF elevates 6 h compressive strength in ultra-high-performance concrete (UHPC) by 4 MPa, meeting critical specifications for rapid repair applications. Notably, research has predominantly focused on conventional concrete modifications, leaving a significant knowledge gap regarding SF’s dual-phase influence on both early-age engineering performance and microstructural evolution in FSS systems. Moreover, the development of high-performance cementitious composites necessitates the synergistic optimization of supplementary cementitious material (SCM) proportioning and alkaline activation mechanisms [25]. Recent advances by Alonso et al. [26] and Fang et al. [27] have revealed that SF-incorporated systems activated by NaOH-Na_2_SO_4_ solutions achieve peak mechanical performance at an optimized NaOH/Na_2_SO_4_ mass ratio of 2.5. Paradoxically, while NaOH-based systems demonstrate superior activation efficiency—as evidenced by Liu et al. [18] reporting 11.72% and 13.44% strength improvements at 7/28 days in geopolymerized marine sediments with 2% NaOH additions—their practical implementation is constrained by multiple factors, including elevated costs, handling risks associated with extreme alkalinity, material corrosivity, rapid setting characteristics, and long-term stability concerns. In this context, quicklime (CaO) emerges as an economically viable alternative. Through hydration-generated Ca(OH)_2_, it simultaneously provides the high-pH environment and soluble Ca^2+^ ions required for sustained pozzolanic reactions while demonstrating superior compatibility with FSS formulation requirements [28].

This study presents a novel, integrated approach for optimizing dredged sediment FSS under quicklime-activated alkaline conditions, thus simultaneously enhancing flowability retention and accelerating early-age strength development—capabilities that have not been addressed together in previous research. SF replacement levels (mass percentage of cement replacement; i.e., 0%, 4%, 8%, and 12%) and water–solid ratios (WSR, defined as the ratio of water mass to the total mass of materials; i.e., 0.525, 0.55, and 0.575) were systematically varied and a comprehensive suite of tests was employed, including time-dependent rheological measurements to track flowability over the critical construction window; unconfined compressive strength and direct shear tests to quantify early mechanical gain; and SEM/XRD analyses to reveal the micro/nano-reaction pathways underpinning performance. Anchored in applied materials science, this study deepens our theoretical understanding of pozzolanic reaction control and establishes empirically validated mix design guidelines for marine scour remediation and other rapid-repair applications.

## 2. Materials and Methods

### 2.1. Test Materials

**Figure 1:**
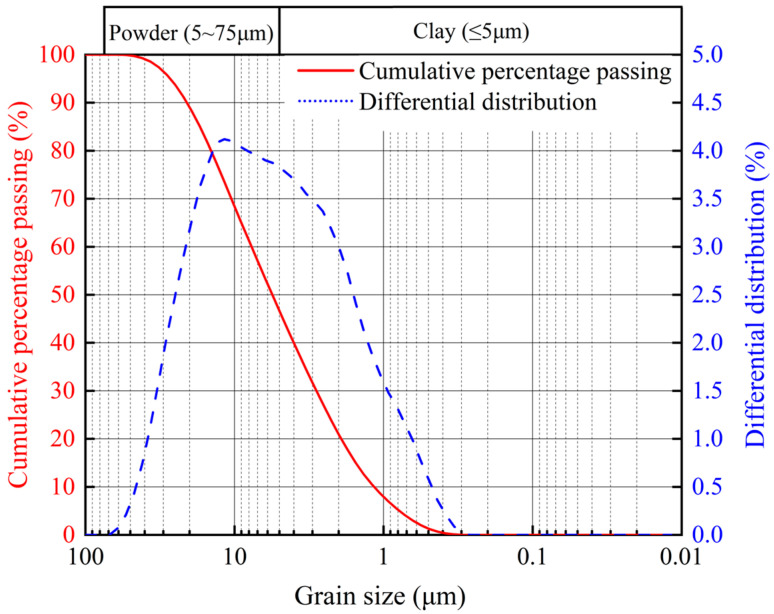
Grain size distribution curve of the dredged sediment.

The dredged sediment used in this study (Figure 1) was collected from a project in Zhejiang Province, China. The material is gray, exhibits low strength and high compressibility, and contains organic matter. Its fundamental physical indices are summarized in Table 1. The natural water content of the sediment exceeds its liquid limit, and the natural porosity ratio is greater than 1.5. According to the soil classification criteria outlined in the Chinese standards [29], the soil sample is classified as sludge. The particle size distribution of the dredged soil was measured using the Biotek-2600 laser particle size analyzer. Figure 1 reveals that the clay particle content (≤5 μm) is 46.69%, while the powder particle content (5~75 μm) is 53.31%.

The materials employed in the curing process encompass cementitious materials, including cement, SF, and quicklime, as well as additional water-reducing agents. In engineering practice, the curing agent dosage typically ranges from 5% to 20% of the dry soil mass. Insufficient cement content can result in inadequate strength development in the FSS. Therefore, the total admixture of curing agents (cement, SF, and quicklime) was fixed at 20% of the dry soil mass for all test specimens.

**Figure 2:**
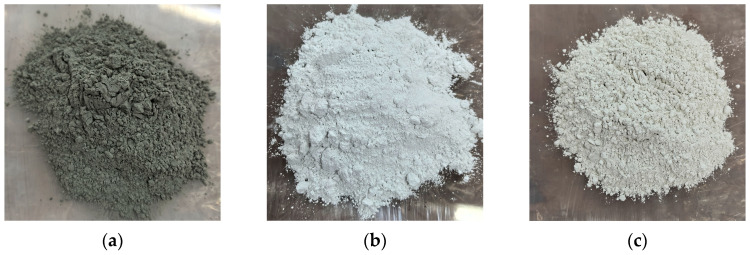
Experimental solidification agents: (**a**) cement; (**b**) silica fume; (**c**) quicklime.

The cement (Figure 2a) was PO42.5 silicate cement from Huai’an Conch Cement Co., Ltd. (Huai’an, China). It had a specific surface area of 360 m^2^/kg and a standard consistency water consumption of 28.9%.

The SF (Figure 2b) was produced from Henan Platinum Run Foundry Materials Co., Ltd. (Zhengzhou, China), with a specific surface area of 22,100 m^2^/kg, SiO_2_ content of over 98%, scorch reduction of 1.48%, and alkali content of 0.18%.

The quicklime (Figure 2c) was produced from Huangbaozhu Daily Chemical Products Co. (Fenyi County, China), with a purity of ≥95%. To construct an efficient alkali excitation environment, the quicklime dosage was set at 2% (percentage of dry soil) in this experiment.

A liquid polycarboxylic acid water-reducing agent, produced by Zhejiang Jiaogong Group (Hangzhou, China), was utilized to ensure the adequate flowability of the FSS while minimizing water secretion. The solid content of the agent was determined to be 20% using the cauterization method. The impact of this water-reducing agent on the strength and rheology of FSS was investigated, and the optimal dosage was 2% of the total amount of cementitious materials.

Tap water from the laboratory was utilized as mixing water in all experiments. The chemical compositions of the three types of cementitious materials are presented in Table 2.

### 2.2. Mixture Proportion

In practical engineering applications, the flowability of FSS typically ranges from 160 mm to 220 mm. Preliminary tests on mixtures with WSRs between 0.40 and 0.60 revealed that restricting WSR to 0.525–0.575 achieves dual objectives: satisfying pumping requirements while preventing slurry segregation characteristics at higher ratios. Accordingly, three WSRs (i.e., low = 0.525; middle = 0.55; and high = 0.575) were selected to balance pumpability and mixture homogeneity. Similarly, the literature indicates that SF replacement rates above 15% adversely affect mechanical performance [30]; therefore, SF substitution levels of 0%, 4%, 8%, and 12% (designated as SF0, SF4, SF8, and SF12, respectively) were adopted to explore the beneficial range below this threshold. This study evaluates how these WSR and SF substitution levels (Table 3) modify the engineering behavior of dredged-sediment-based FSS. The proportions of individual curing agents are expressed as percentages relative to the total mass of the curing agents.

### 2.3. Preparation of Dredged-Sediment-Based FSS

The specimen preparation was conducted according to the Specification for Mix Proportion Design of Cement Soil (JGJ/T 233-2011) [31] in China. Compressive specimens were cast in cubic molds with dimensions of 70.7 mm × 70.7 mm × 70.7 mm, while the straight shear test samples were obtained by cutting the vibrated cubic specimens using a ring cutter with a diameter of 61.8 mm and a height of 20 mm.

**Figure 3:**
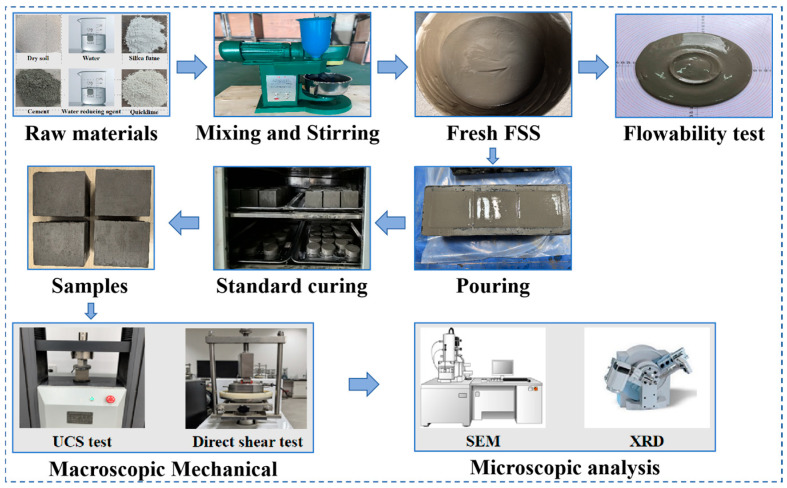
Preparation flowchart for dredged-sediment-based FSS.

Prior to specimen preparation, the dredged sediment was dried in an oven at 65 °C to a constant weight and subsequently pulverized and sieved through a 2 mm sieve for spare parts. A cementitious sand mixer was utilized to prepare the FSS. To ensure that the test soil samples and the curing agent were thoroughly mixed, the solid was initially introduced into the mixer and mixed at a low speed for two minutes. Subsequently, water was added to the mixture to continue the mixing process for five additional minutes. The resulting mixture was carefully poured into the cube molds in layers, with the surface smoothed with a scraper. The specimen was then vibrated at a uniform speed using a vibrating table. Specimens underwent an initial natural curing process period of 36 to 48 h, after which they were demolded and transferred to a standard curing box. The curing conditions were maintained at a temperature of 20 ± 2 °C and a relative humidity of no less than 95% until the specimens reached the specified curing age. The procedure for FSS preparation is illustrated in Figure 3.

### 2.4. Test Methods

#### 2.4.1. Flowability Test

The flowability test is a critical index for evaluating the working performance of FSS. The value and duration of the flow state directly determine the application scope of the project. According to the specifications outlined in the “Test Method of Air Mortar” (JHS A313-1992) [32], the experimental apparatus consists of an acrylic cylinder measuring 80 mm in height and diameter, along with a square test plate that spans 400 mm in length. These components were utilized in the present study. Prior to the test, the cylinder was uniformly coated with petroleum jelly to minimize the effect of adhesion between the slurry and the cylinder wall. The treated cylinder was positioned centrally on the test plate and subsequently subjected to a gradual injection of a well-mixed FSS until the cylinder was fully saturated. Thereafter, the liquid surface was meticulously smoothed using a spatula to ensure its alignment with the top surface of the cylinder. Subsequently, the cylinder was elevated at a uniform velocity, and following a one-minute interval of repose, the maximum slump diameter and its vertical diameter were recorded on the base plate scale. The arithmetic mean of these two values was then calculated as the mobility of the FSS.

#### 2.4.2. Unconfined Compressive Strength (UCS) Test

Unconfined compressive strength is a critical metric for evaluating the mechanical properties, bearing capacity, and stability of FSS. This test was selected based on the standard maintenance protocol, which includes 3-, 7-, and 28-day test block durations. The test procedure was conducted according to the specifications outlined in the Standard for Test Method of Basic Properties of Construction Mortar (JGJ/T 70-2009) [33]. The loading was performed using “BOVEN50Tpro”, a microcomputer-controlled electronic universal testing machine produced by Shanghai Platinum Temperature Instrument Co., Ltd. (Shanghai, China). The loading rate of “BOVEN50Tpro” was 1 mm/min. During the loading process, the computer automatically collected the stress–strain data of the specimen. When the specimen stress reached its peak value, it continued to be loaded for a predetermined period until the conclusion of the test. At that point, the specimen was automatically reset, and the peak value was recorded. This recorded peak value is defined as the UCS of the specimen.

#### 2.4.3. Shear Strength Test

Studies have shown that scour occurs when hydraulic shear stress exceeds a soil’s critical shear stress [34]; therefore, scour resistance is closely related to this threshold, and shear strength is typically governed by cohesion (c) and an internal friction angle (φ). Accordingly, measuring the shear strength of FSS evaluates its scour resistance. In this study, the direct shear method was used to determine c and φ in specimens cured for 7 days and 28 days, following the procedures specified in the Standard for Soil Test Method (GB/T 50123-2019) [35] in China. The test equipment utilized was the “HK-PZ-06F”, a fully automatic straight shear apparatus produced by Beijing Huajan Technology Co., Ltd. (Beijing, China), and the test was carried out under four kinds of vertical loads (100, 200, 300, and 400 kPa), with the shear rate in the shear box set at 0.8 mm/min and shear displacement at 6 mm.

#### 2.4.4. Microscopic Parameter Testing

Subsequently, small pieces of the samples were extracted from the specimens following the unconfined compressive test. These samples were then immersed in anhydrous ethanol for 24 h to terminate the hydration reaction. Thereafter, the samples were subjected to a drying process in a vacuum oven at 60 °C for 48 h. Subsequent to the drying process, a subset of the specimens was ground into a powder using a mortar and sieved through a 200-mesh screen. These specimens were then stored under vacuum conditions for X-ray diffraction (XRD) testing. The remaining specimens, each measuring approximately 1 cm^3^ and featuring a fresh cross-section, were meticulously polished with sandpaper until they were perfectly flat. These specimens were then stored under vacuum conditions for scanning electron microscopy (SEM).

A Japanese “Rigaku SmartLab SE” X-ray diffractometer (Tokyo, Japan) was utilized for the XRD analysis test, employing a copper target with a scanning speed of 1°/min and a scanning range from 5° to 90° to systematically analyze the composition of the physical phase of the FSS.

Microstructural observations of FSS were conducted using an American “FEI Scios 2 HiVac” scanning electron microscope (Thermo Scientific, Waltham, MA, USA). Secondary electron imaging was employed at magnifications of ×5000 and ×20,000. To circumvent the discharge phenomenon engendered by charge aggregation on the sample surface, a gold film was uniformly coated on the sample surface prior to the test.

## 3. Results and Discussion

### 3.1. Flowability

#### 3.1.1. Initial Flowability

**Figure 4:**
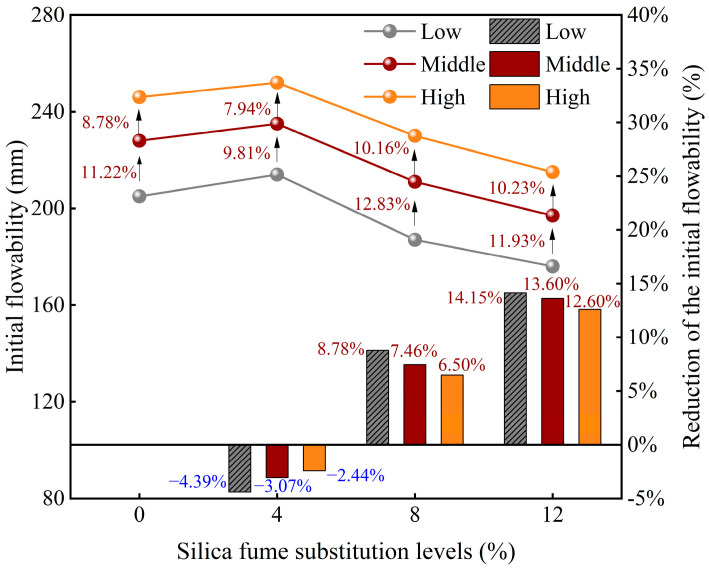
Initial flowability of FSS with different levels of SF substitution.

Figure 4 illustrates the variation in the initial flowability of FSS with different levels of SF substitution. The results show that the initial flowability reaches its maximum at a 4% SF substitution, corresponding to the optimal working performance. However, as the SF substitution increases beyond 4%, the initial flowability demonstrates a gradually decreasing trend. Specifically, the increased initial flowability observed for SF4 (4% SF substitution) compared with SF0 (the control group) was within 5%. In contrast, the reduced initial flowability for SF8 (8% SF substitution) and SF12 (12% SF substitution) fell within ranges of 5–10% and 10–15%, respectively. These findings suggest that incorporating moderate amounts of SF enhances the workability of FSS, whereas excessive SF content diminishes the workability of soil. The improved flowability at low SF substitution levels can be attributed to the “ball effect” of SF’s spherical and smooth particles, reducing friction and enhancing lubricity [36]. Moreover, the high specific surface area of SF promotes particle dispersion and water adsorption, leading to the formation of a hydration film that further improves flowability [37]. Furthermore, the fine particle size helps fill pores and reduce porosity, thereby enhancing rheology properties [38]. However, SF contents above 8% substantially increase surface area and water demand, reducing available free water and increasing viscosity. Higher SF contents also enhance particle friction and raise yield stress, further diminishing flowability [39].

Figure 4 also shows the impact of the WSR (i.e., low, middle, and high) on the initial flowability of FSS. Under the same SF substitution condition, the initial flowability was significantly enhanced with an increase in WSR. Specifically, an increase in the WSR from 0.525 to 0.55 enhanced the initial flowability by 11.22% to 12.83%, depending on the SF substitution. A further increase in the WSR to 0.575 led to an approximate 20% increase in the initial flowability, underscoring the pivotal role of the WSR in governing flow performance. This phenomenon can be attributed to the increased amount of free water at a high WSR, which promotes better dispersion and more uniform particle arrangement. Additionally, the formation of a continuous water film on particle surfaces reduces internal friction and contact resistance, thereby improving the flow performance of the FSS [40].

#### 3.1.2. Time-Dependent Characteristics of Flowability

Maintaining prolonged flowability not only provides a sufficient window for the construction of FSS but also helps to expand its application domain. Consequently, this study measures the flowability of FSS at 0, 30, 60, 90, and 120 min to investigate the time-dependent evolution of rheological performance during the early construction stage (within 2 h), providing critical data for defining the optimal construction window and optimizing material properties. To ensure slurry homogeneity, each specimen was remixed before testing.

**Figure 5:**
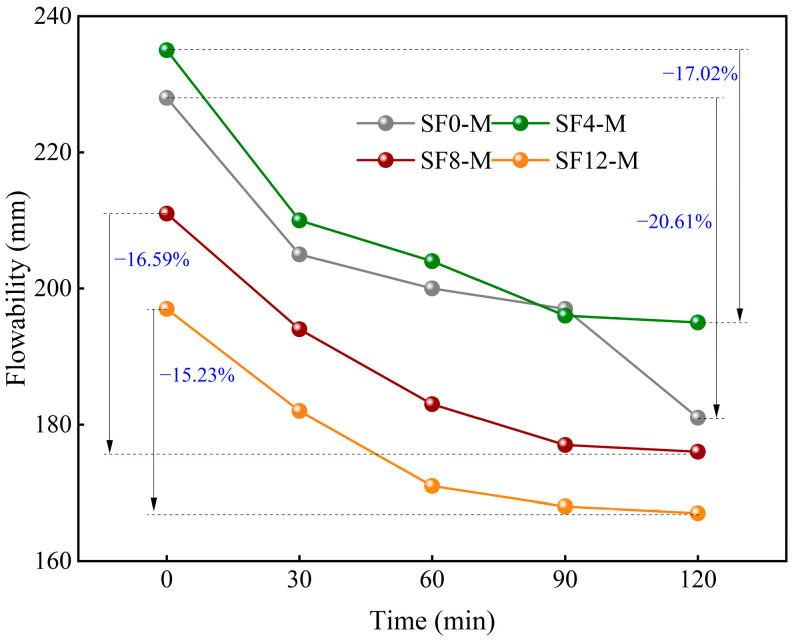
Time-dependent flowability of FSS under different SF substitutions.

**Figure 6:**
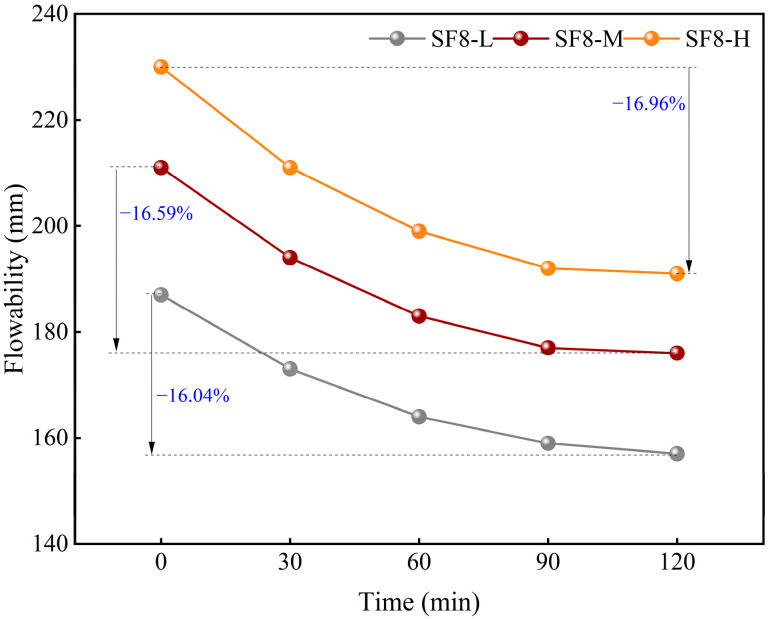
Time-dependent flowability of FSS under different WSRs.

Figure 5 and Figure 6 demonstrate the effects of SF substitution and variations in the WSR on the time-dependent flowability of FSS. The results indicate that the flowability of FSS decreases markedly over time. Specifically, the flowability of FSS without SF replacement (SF0-M) exhibits decreases of 23, 28, 31, and 47 mm after 30, 60, 90, and 120 min, respectively. The flowability of FSS with SF substitution levels of 8% (SF8-M) exhibits decreases of 17, 28, 34, and 35 mm, respectively. This finding suggests that SF replacement substantially influences the characteristics of the time-dependent flowability of FSS. In the group without SF replacement (SF0), the flowability of the FSS decreased rapidly from 0 to 30 min, and the loss rate of the flowability was close to 10%. The decrease from 30 to 90 min slowed down, and the loss rate of the flowability was about 12% and 14% in 60 and 90 min, respectively. However, the decrease in the section from 90 to 120 min had a second surge, and the loss rate of the flowability exceeded 20%. By contrast, the group utilizing SF replacement cement (SF4, SF8, and SF12) exhibited a comparable trend to the group not employing such cement during the 0~90 min interval. However, during the 90~120 min period, the decline rate did not demonstrate a surge; instead, it exhibited a more gradual decline. Furthermore, comparing 2 h flowability loss rates, SF0 exhibited higher losses than SF4, SF8, and SF12, and these losses decreased as SF content increased. This finding suggests that incorporating SF enhances the flowability retention capacity of FSS during the early construction stage (within 2 h), corresponding to the extension of the construction window. This phenomenon can be attributed to the optimization of particle grading using SF, which effectively reduces the specific surface area of cementitious materials and inhibits the early-age hydration activity of C3S/C2S by filling the gaps in the mixed particles and enhancing the stacking compactness. As the SF substitution increases, the particle-stacking compactness also increases, and the hydration reaction slows down more pronouncedly [41].

Notably, the influence of the WSR on the time-dependent evolution of flowability manifests chiefly in the absolute spread values (Figure 6). While the attenuation curves for different WSRs exhibit similar overall shapes, the flowability loss rate at each time point increases linearly with the WSR. Specifically, for the SF8 series, the 2h flowability loss rates of SF8-L, SF8-M, and SF8-H are 16.04%, 16.59%, and 16.96%, respectively. This linear relationship confirms the fundamental role of WSR in regulating the rheological behavior of FSS.

### 3.2. Unconfined Compressive Strength (UCS)

**Figure 7:**
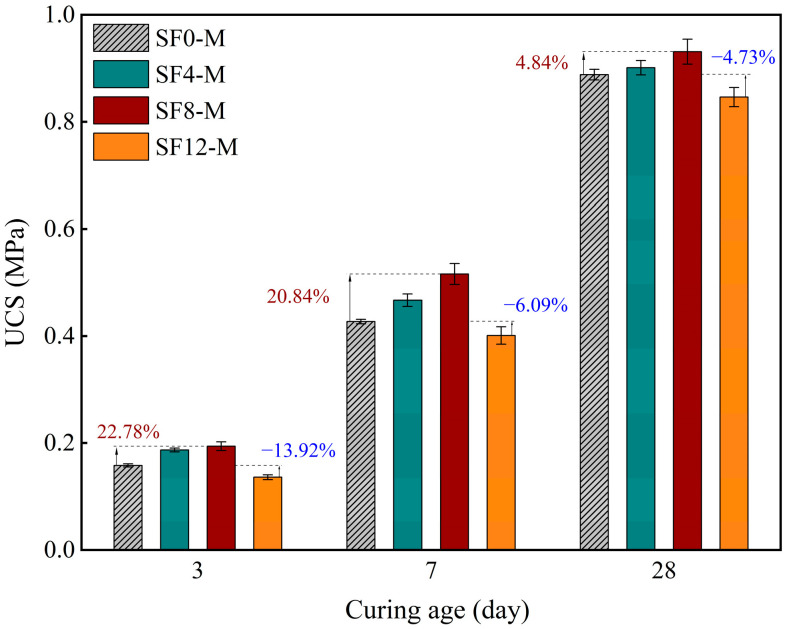
UCS of FSS with varying SF substitution rates at 3, 7, and 28 days of curing.

Figure 7 illustrates variations in the USC of FSS with different levels of SF substitution after 3, 7, and 28 days of curing at a WSR of 0.55 (i.e., SF0-M, SF4-M, SF8-M, SF12-M). The results show that the UCS of FSS exhibited an initial increase, followed by a subsequent decrease, with increasing substitution levels. SF8-M (8% SF substitution, WSR = 0.55) demonstrated optimal performance, exhibiting 22.78%, 20.84%, and 4.84% higher strengths than the control group (SF0-M) at 3, 7, and 28 days, respectively. This result indicates that the volcanic ash effect of SF significantly promotes early-age hydration and accelerates the early-age strength development of FSS under the quicklime–alkali-excited system. SF provides ample reactive SiO_2_, while the alkaline exciter quicklime hydration generates calcium hydroxide, thereby establishing the requisite alkaline environment and reactants for the formation of gels, such as C-S-H, and accelerating the hydration process of FSS [42].

However, when the replacement amount of SF exceeded 8% (SF12-M), the UCS of FSS decreased instead, falling by 13.92%, 6.09%, and 4.73% relative to the control group (SF0-M) at 3, 7, and 28 days, respectively. This observation suggests that the effectiveness of SF in promoting FSS hydration is significantly influenced by the replacement amount of SF. The primary factor contributing to this phenomenon is the close correlation between the compressive strength of FSS and its pore structure. Specifically, the amorphous SiO_2_ present in SF underwent a reaction with Ca(OH)_2_ under conditions of elevated alkalinity. This reaction led to the secondary generation of C-S-H gel, which subsequently infiltrated the interior of the material’s pore space. The subsequent effects of this process included a reduction in pore size and the number of interconnected pores, particle lapping optimization, and an improvement in the densification of the interface. However, when the amount of SF substitution exceeded a critical threshold value, the solid particles were not arranged uniformly, and the efficiency of reaction product generation and filling decreased. This resulted in an inadequate interparticle bonding interface and a decrease in the overall cohesion of the matrix [43]. Consequently, the structural integrity and mechanical properties of the material were impaired. In summary, there is a threshold effect between the amount of SF substitution and the mechanical properties of FSS. Therefore, it is imperative to determine a reasonable amount of FS substitution to ensure the efficient curing and engineering application of FSS.

**Figure 8:**
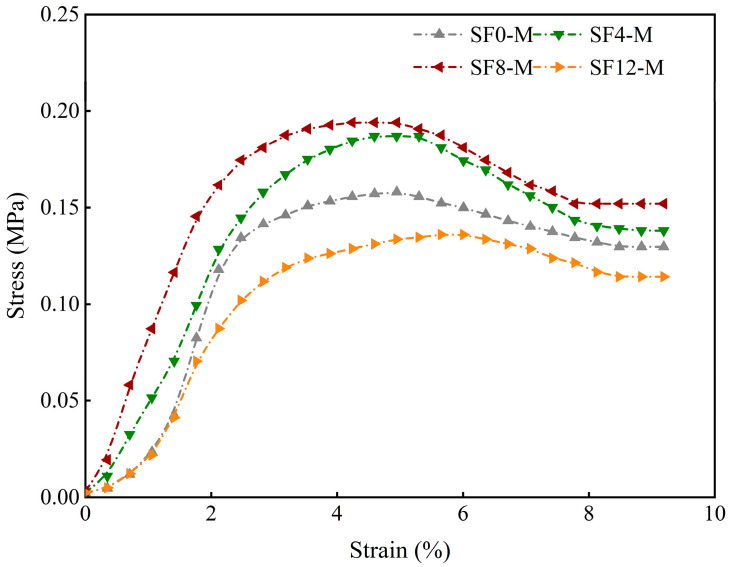
Stress–strain curves of different SF substitution rates at 3 days of curing.

This pronounced threshold effect also manifests in the early-age axial compression stress–strain response. Taking the 3-day curing age (Figure 8) as an example, SF8-M curves shifted upward and leftward relative to the control (SF0-M), indicating significantly enhanced strength and brittleness, while SF12-M curves shifted downward and rightward, reflecting diminished strength, stiffness, and load-bearing capacity.

**Figure 9:**
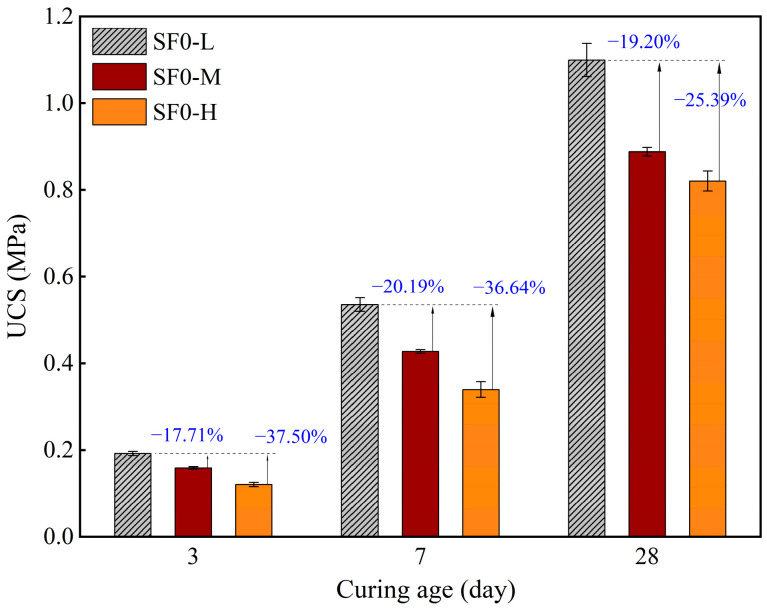
UCS of FSS with varying WSRs at 3, 7, and 28 days of curing.

**Figure 10:**
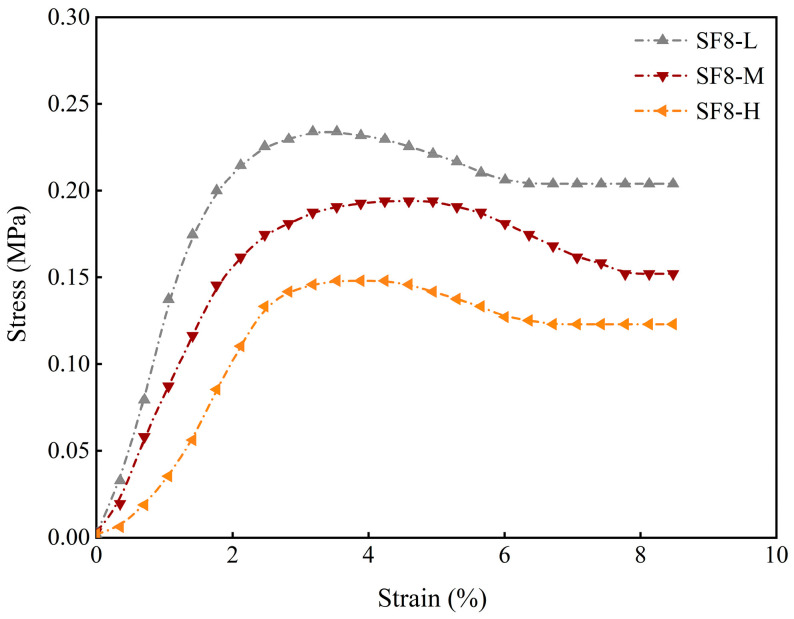
Stress–strain curves of different WSRs at 3 days of curing.

Figure 9 illustrates variations in the UCS of FSS without SF replacement with different WSRs after 3, 7, and 28 days of curing (i.e., SF0-L, SF0-M, and SF0-H). The findings indicated a decline in the UCS of FSS with an increase in the WSR. The UCS values of SF0-M (without SF replacement, WSR = 0.55) and SF0-H (without SF replacement, WSR = 0.575) at 7 days were 20.19% and 36.64% lower, respectively, than that of SF0-L (without SF replacement, WSR = 0.525). This trend corresponds to the stress–strain curve of FSS (Figure 10) moving to the lower right with an increased water-to-solid ratio and is consistent with the experimental law reported by Abbas and Qureshi [44] that WSR-UCS is negatively correlated in cement-based systems. This phenomenon is primarily due to the impact of the WSR on the microstructure of the matrix and the subsequent generation of cementitious products. An elevated WSR results in an augmented dilution effect in the paste, an increased porosity within the curing system, a limitation on the effective generation and distribution of C-S-H gels, and the formation of weak interfacial zones. This, in turn, reduces the compactness and mechanical strength of the matrix [45]. Therefore, the study of the UCS characteristics of FSS with different WSRs is crucial to optimize the mix proportion. This optimization is necessary to ensure sufficient rheology while enhancing the mechanical properties and achieving the balanced optimization of the material properties.

### 3.3. Shear Strength

**Figure 11:**
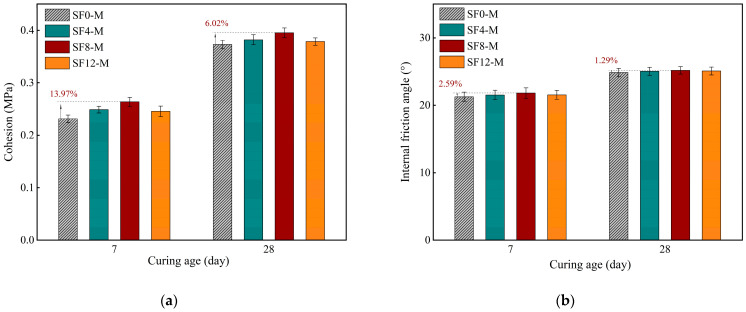
Direct shear parameters of FSS with different SF substitution at 3, 7, and 28 days of curing: (**a**) cohesion; (**b**) internal friction angle.

Figure 11 shows variations in the cohesion (*c*) and internal friction angle (*φ*) of FSS with different SF replacements after 3, 7, and 28 days of curing at a WSR of 0.55 (i.e., SF0-M, SF4-M, SF8-M, and SF12-M). The cohesion first increases and then decreases with the increased SF replacement. SF8-M performs best, 13.97% and 6.02% higher than the control group (SF0-M) at 7 and 28 days, respectively. In contrast, the internal friction angle changes less but still shows a similar trend, which is 2.59% and 1.29% higher in SF8-M than in the control group. The test results show that the improved early-age shear strength of FSS caused by SF is dominated by cohesion, which is achieved by enhancing the bonding ability between particles rather than significantly improving the interface friction characteristics. However, when the SF replacement amount is increased to 12% (SF12-M), the cohesion and internal friction angle of the sample compared with SF8-M show different degrees of decrease, indicating that excessive SF adversely affects the shear resistance of FSS. The main reason is that when the SF replacement amount is too large, the supply of Ca(OH)_2_ is insufficient, and some SF particles cannot be fully hydrated and transformed into inert filling particles. In turn, this affects the formation of C-S-H gel and its uneven distribution, forming local weak areas.

**Figure 12:**
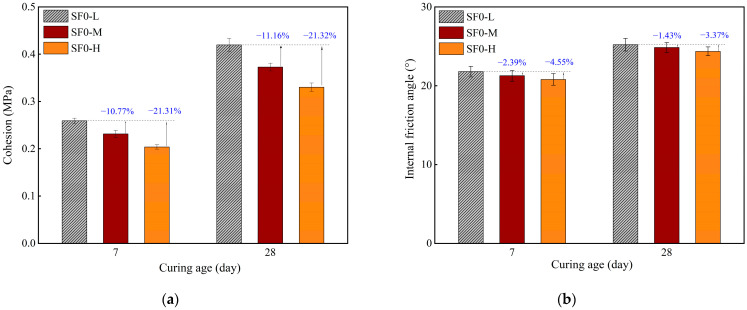
Direct shear parameters of FSS with different WSRs at 3, 7, and 28 days of curing: (**a**) cohesion; (**b**) internal friction angle.

Figure 12 shows variations in the cohesion (*c*) and internal friction angle (*φ*) of FSS without SF with different WSRs after 3, 7, and 28 days of curing (i.e., SF0-L, SF0-M, and SF0-H). The results show that with the increased WSR, the cohesion and internal friction angle of the sample show a downward trend, of which *c* decreases more significantly. SF0-M and SF0-H are 10.77% and 21.31% lower than the low SF0-L at 7 days. The main cause is the effect of the WSR on matrix porosity and shear failure mode. A high WSR enhances the dilution effect of slurry, increases porosity, weakens the bridging effect of C-S-H gels, reduces the particle bonding capacity, and thus significantly reduces cohesion. At the same time, the number of weak interface areas increases, and the stress transfer between particles is hindered, decreasing the internal friction angle. Therefore, to ensure the working performance of FSS, it is very important to optimize the WSR to enhance the bonding effect and stress transfer capacity between particles, thereby improving the shear resistance.

### 3.4. Microscopic Analysis

#### 3.4.1. X-Ray Diffraction (XRD)

**Figure 13:**
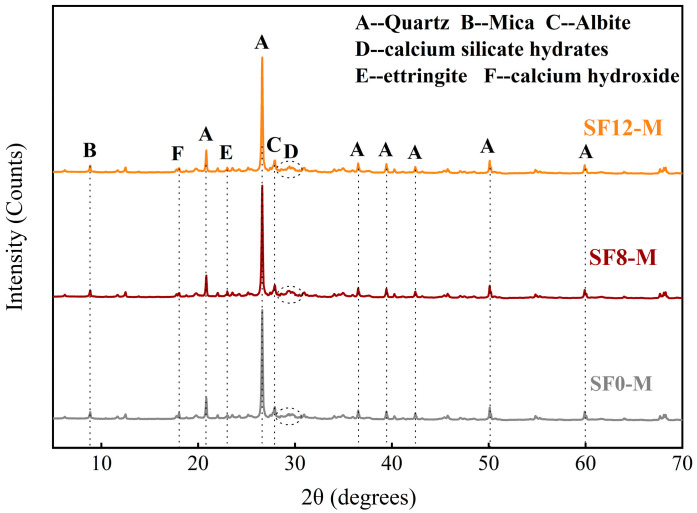
X-ray diffraction pattern of FSS with different SF substitutions.

**Figure 14:**
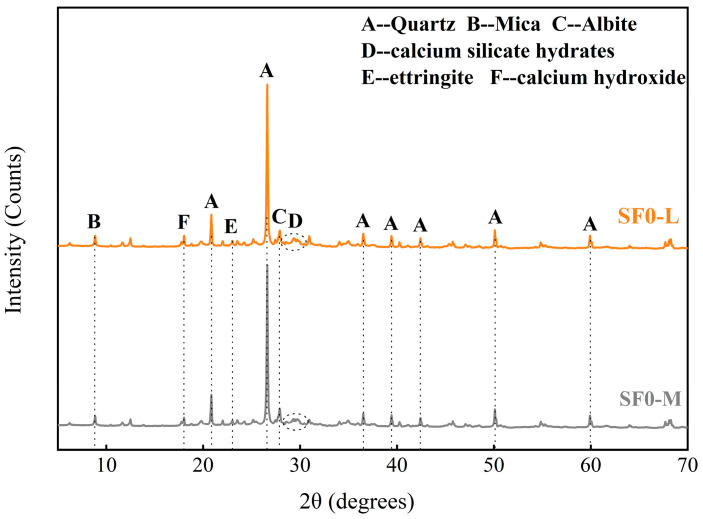
X-ray diffraction pattern of FSS with different WSRs.

XRD analysis was conducted to elucidate the effects of SF substitution and WSR on hydration product formation and phase assemblages in FSS. Figure 13 and Figure 14 display diffraction patterns for SF-free samples (SF0-L and SF0-M) and SF-substituted samples (SF8-M and SF12-M) after 3 days of curing. While all groups share similar primary crystalline phases (quartz, mica, albite, calcium silicate hydrates (C-S-H), ettringite (AFt), and calcium hydroxide (Ca(OH)_2_)), significant quantitative differences emerge in key hydration products, mainly judged by the relative intensities of the key diffraction peak. Observing the broad hump at 2θ ≈ 29°, we can see that the diffraction peak intensities of the three groups are SF8-M > SF12-M > SF0-M, confirming that the pozzolanic effect of silica fume and calcium hydroxide generates additional C-S-H gel in the early curing stage. SF12-M exhibits weaker hump intensity than that of SF8-M owing to the presence of unreacted SF and less cement content in SF12-M. SF0-M displays markedly higher calcium hydroxide peak intensity than SF8-M and SF12-M, validating the Ca(OH)_2_ consumption caused by SF’s pozzolanic reaction during early hydration. Additionally, SF8-M exhibits stronger ettringite peaks than SF0-M; it may be that moderate alkalinity enhances AFt formation by promoting aluminum dissolution, whereas excessive Ca(OH)_2_ in SF0-M inhibits it. Finally, variations in the WSR mainly affect absolute peak intensities, in line with their observed impact on early-age mechanical performance.

#### 3.4.2. Scanning Electron Microscopy (SEM)

**Figure 15:**
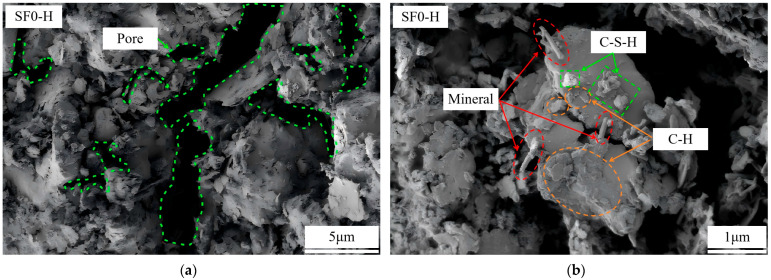

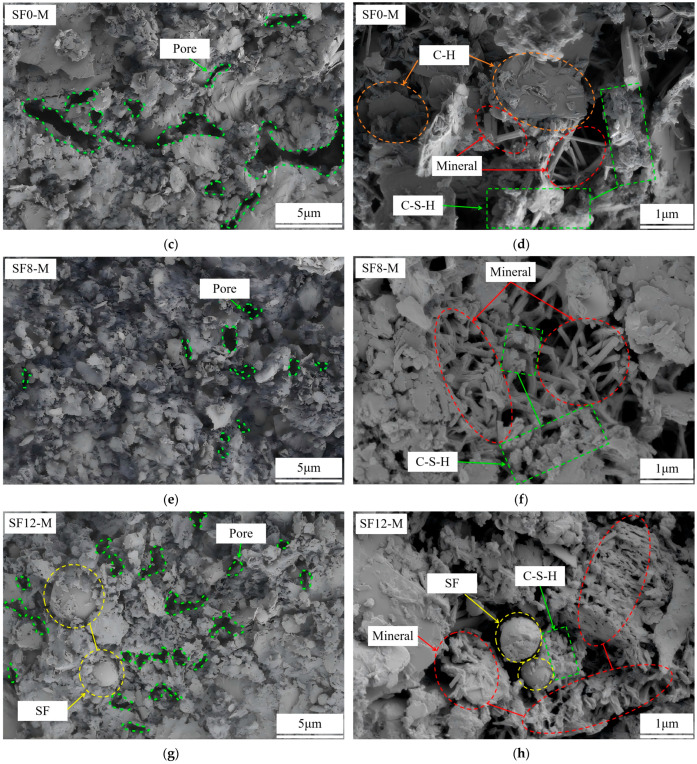
SEM of FSS with different mix proportions: (**a**) 0% SF substitution, WSR = 0.575, ×5000; (**b**) 0% SF substitution, WSR = 0.575, ×20,000; (**c**) 0% SF substitution, WSR = 0.55, ×5000; (**d**) 0% SF substitution, WSR = 0.55, ×20,000; (**e**) 8% SF substitution, WSR = 0.55, ×5000; (**f**) 8% SF substitution, WSR = 0.55, ×20,000; (**g**) 12% SF substitution, WSR = 0.55, ×5000; (**h**) 12% SF substitution, WSR = 0.55, ×20,000.

To analyze the regulatory effect of SF substitution and the WSR on the microstructure of FSS, representative groups (i.e., SF0-M, SF0-H, SF8-M, and SF12-M) were examined via SEM at ×5000 and ×20,000 magnifications (Figure 15). The ×5000 magnification can fully reflect the overall structural characteristics of the soil, such as the distribution of soil particles, the overlap form, and pore size, while the ×20,000 magnification is convenient for observing the morphology of cementitious materials, mineral crystal size, and its connection method in local areas.

The results indicate that under the same WSR, SF substitution has a pronounced impact on the microstructure of FSS. Specifically, many plate-like Ca(OH)_2_ crystals and a small number of acicular–columnar minerals and C-S-H gels were generated in the FSS without SF substitution (SF0-M). These hydration products were mainly distributed on the surface of soil particles or filled in the pores. However, in local areas, the pore size was large (with some reaching over 5 μm), indicating that although the hydration products were generated, there was a lack of sufficient cementation, resulting in a relatively loose overall structure. Conversely, FSS with 8% SF substitution (SF8-M) showed obvious structural densification characteristics, with many acicular–columnar minerals and C-S-H gels uniformly filling the pores, achieving close overlap through multiple contact modes (such as face-to-face and edge-to-face) and constructing a continuous and dense three-dimensional network structure, such that the pore size was mainly controlled below 2 μm. This shows that an appropriate amount of SF substitution helps to fully stimulate the volcanic ash reaction and generate fine and evenly distributed hydration products. These products can not only effectively fill the pores but also closely combine with soil particles through various contact forms, thereby optimizing the pore structure and enhancing the continuity of the overall structure. This microscopic densification structure is the key to improving the mechanical properties of FSS. However, in the FSS with a higher SF substitution (SF12-M), although the formation of acicular–columnar minerals and C-S-H gel can still be observed, some unreacted SF particles are mixed between the two, hindering the continuity of the cementitious products, resulting in obvious spatial heterogeneity in the microstructure. The pore sizes, while reduced compared with SF0-M, were nonetheless slightly larger than those of SF8-M. This shows that adding excessive SF may reduce the utilization rate of active components in the system due to incomplete reaction and particle agglomeration effects, destroy the original cementing network, and verify the result of reduced mechanical properties.

In addition, we compared the micromorphology of FSS without SF substitution under different WSRs (i.e., SF0-M and SF0-H). The results show that both groups of samples generate many plate-like Ca(OH)_2_ crystals and a small number of acicular–columnar minerals and C-S-H gels, and there is no significant difference in the types or quantities of their hydration products. However, the internal structure of SF0-H is obviously looser, the number of pores is significantly increased, the overlap between particles is not obvious, the pore size is generally larger, and its maximum pore size is about 1.5 times that of SF0-M. This is mainly due to the significant increase in the free water content in the reaction medium under high WSR conditions, making the generated hydration products more dispersed in space; they fail to fully play the role of binder, thereby reducing the physical cross-linking effect between the hydration products and then damaging the overall structural density.

In summary, SF substitution and the WSR have significant and complex regulatory effects on the microstructure of FSS, which is mainly reflected in the kinetics of the hydration reaction and the construction of the cementation network. An appropriate SF substitution and WSR can optimize the synergistic effect of cement, quicklime, and SF in the reaction system; promote the full progress of the volcanic ash reaction; and generate C-S-H gels and other hydration products that can fill pores and form a continuous load-bearing skeleton at the microscopic scale, thereby simultaneously improving compressive and shear strength. On the contrary, excessive SF substitution or a too-high WSR will lead to the uneven distribution of hydration products, fractures in the cementing network, and increased porosity, forming a local weak interface area, which is not conducive to the performance of mechanical properties.

## 4. Conclusions

This study compared the early-age engineering properties (flowability, UCS, and shear strength) and microstructure of dredged-sediment-based FSS under quicklime–alkali activation conditions with different WSRs and SF cement substitution levels. We drew the following conclusions:

SF substitution significantly mitigates flowability loss during the 90–120 min placement window, extending the operational period by over 0.5 h. Flowability decay over 2 h increases linearly with the water–solid ratio (WSR), with SF8-L, SF8-M, and SF8-H exhibiting 16.04%, 16.59%, and 16.96% losses, respectively. These findings demonstrate that both SF substitution and the WSR are pivotal in maintaining pumpable rheology for self-leveling scour pit filling.

An 8% SF substitution at WSR = 0.55 delivers the greatest enhancement in the early-age strength of FSS, boosting 3-day UCS by 22.78% and increasing the 7-day cohesion and internal friction angle by 13.97% and 2.59%, respectively. SEM and XRD analyses confirmed that SF’s pozzolanic reaction produces additional C-S-H gel and promotes ettringite formation, constructing a dense three-dimensional network that underpins improved mechanical resistance to hydraulic shear. This directly eases the vulnerability of conventional FSS to erosion-induced cracking before sufficient curing.

A pronounced threshold effect governs optimization efficacy across properties. Flowability peaked at 4% SF, whereas mechanical performance was maximized at 8% SF before declining due to unreacted particle accumulation. Elevated WSR similarly degrades integrity by inducing heterogeneous hydration and weak interfacial zones. Though real-world hydraulic erosion simulation will be the focus of a future study, the described threshold mechanism provides critical theoretical guidance for balancing flow retention and erosion resistance in marine environments.

## Figures and Tables

**Table 1:** Basic physical properties of the dredged sediment.

Natural Water Content/%	Natural Porosity Ratio	Liquid Limit/%	Plastic Limit/%	Plasticity Index
67.2%	1.81	46.2%	24.2%	22

**Table 2:** Chemical composition of experimental solidification agents (%).

Material	SiO_2_	CaO	Al_2_O_3_	MgO	Fe_2_O_3_
Cement	22.31	54.36	9.76	1.01	3.13
Silica fume	98.1	/	0.19	/	0.12
Quicklime	/	95	/	/	/

**Table 3:** Mix proportion and experimental design of WSR.

No.	Water–Solid Ratio (WSR)	SF Replacement Rate/%	Proportions of Solidification Agent Components/%		
			Cement	SF	Quicklime
SF0-L	0.525	0	90	0	10
SF0-M	0.55	0	90	0	10
SF0-H	0.575	0	90	0	10
SF4-L	0.525	4	86.4	3.6	10
SF4-M	0.55	4	86.4	3.6	10
SF4-H	0.575	4	86.4	3.6	10
SF8-L	0.525	8	82.8	7.2	10
SF8-M	0.55	8	82.8	7.2	10
SF8-H	0.575	8	82.8	7.2	10
SF12-L	0.525	12	79.2	10.8	10
SF12-M	0.55	12	79.2	10.8	10
SF12-H	0.575	12	79.2	10.8	10

## Data Availability

The original contributions presented in this study are included in the article. Further inquiries can be directed to the corresponding author.

## References

[1] Wang H., Wang Z.W., Yang W.C., Jia H.L., You Z.J. (2025). Characterization, pollution, and beneficial utilization assessment of dredged sediments from coastal ports in China. Mar. Pollut. Bull..

[2] Beljin J., Arsenov D., Slijepcevic N., Maletic S., Dukanovic N., Chalot M., Zupunski M., Pilipovic D.T. (2023). Recycling of polluted dredged sediment–Building new materials for plant growing. Waste Manag..

[3] Bian X., Ren Z.L., Zeng L.L., Zhao F.Y., Yao Y.K., Li X.Z. (2024). Effects of biochar on the compressibility of soil with high water content. J. Clean Prod..

[4] Ding J.W., Wan X., Jiao N., Zhang S., Chen W.H. (2024). Collaborative effects of red mud and phosphogypsum on geotechnical behavior of cement-stabilized dredged clay. Bull. Eng. Geol. Environ..

[5] Song D.B., Chen W.B., Yin Z.Y., Shi X.S., Yin J.H. (2023). Recycling dredged mud slurry using vacuum-solidification combined method with sustainable alkali-activated binder. Geotext. Geomembr..

[6] Zentar R., Wang H.W., Wang D.X. (2021). Comparative study of stabilization/solidification of dredged sediments with ordinary Portland cement and calcium sulfo-aluminate cement in the framework of valorization in road construction material. Constr. Build. Mater..

[7] Feng D.z., Liang B., He X.X., Yi F., Xue J.F., Wan Y., Xue Q. (2023). Mechanical properties of dredged soil reinforced by xanthan gum and fibers. J. Rock Mech. Geotech. Eng..

[8] Zhang S.W., Zhu Q.C., Devries W., Ros G.H., Chen X.H., Muneer M.A., Zhang F.S., Wu L.Q. (2023). Effects of soil amendments on soil acidity and crop yields in acidic soils: A world-wide meta-analysis. J. Environ. Manag..

[9] Huo P.S., Fu X.R., Che Z., Liang J.B., Li D.X., Liu Y.L., Lyu S. (2024). Simultaneous improvement of water/fertility retention and physical properties of dredged sediment using a novel composite amendment. Water Air Soil Pollut..

[10] Adazabra A.N., Viruthagiri G., Atingabono J. (2023). Developing fired clay bricks by incorporating scrap incinerated waste and river dredged sediment. Process Saf. Environ. Protect..

[11] Deng X.T., Jian R.L., Chen S., Wang X.S., Wan C., Xue Y.J., Wang T. (2023). Killing two birds with one stone: Preparation of ceramsite high-strength lightweight aggregate via co-sintering of dredged sediment and municipal solid waste incinerated fly ash. Constr. Build. Mater..

[12] Ghadakpour M., Choobbasti A.J., Kutanaei S.S. (2020). Experimental study of impact of cement treatment on the shear behavior of loess and clay. Arab. J. Geosci..

[13] Ho T.O., Chen W.B., Yin J.H., Wu P.C., Tsang D.C.W. (2021). Stress-strain behaviour of cement-stabilized Hong Kong marine deposits. Constr. Build. Mater..

[14] Dermatas D., Dutko P., Balorda-Barone J., Moon D. (2002). Geotechnical properties of cement treated dredged sediment to be used as transportation fill. Dredging ’02.

[15] Donrak J., Horpibulsuk S., Arulrajah A., Kou H.L., Chinkulkijniwat A., Hoy M. (2020). Wetting-drying cycles durability of cement stabilised marginal lateritic soil/melamine debris blends for pavement applications. Road Mater. Pavement Des..

[16] Liu C., Berard C., Deng L.j. (2023). Engineering behavior of cement-treated stiff clay subjected to freezing/thawing cycles. Cold Reg. Sci. Tech..

[17] Wan X., Ding J.W., Mou C., Gao M.Y., Jiao N. (2024). Role of Bayer red mud and phosphogypsum in cement-stabilized dredged soil with different water and cement contents. Constr. Build. Mater..

[18] Liu J.J., Luo H.P., Lei H.Y., Zheng G., Cheng X.S. (2024). Compressive strength and curing mechanism of alkali-activated geopolymer curing marine silty soft soil. J. Railw. Sci. Eng..

[19] Liu F.Y., Zhu C.G., Yang K.J., Ni J.F., Hai J., Gao S.H. (2021). Effects of fly ash and slag content on the solidification of river-dredged sludge. Mar. Geores. Geotechnol..

[20] Wang C., Jin L.W., Qian Y., Wu Y.J., Liang F.Y. (2025). Investigation of the protection mechanism and failure modes of solidified soil utilized for scour mitigation. Constr. Build. Mater..

[21] Mao Y.Z., Jiao D.W., Hu X., Jiang Z., Shi C.J. (2024). Effect of dispersion behavior of silica fume on the rheological properties and early hydration characteristics of ultra-high strength mortar. Cem. Concr. Compos..

[22] Xi J.Y., Liu J.Z., Yang K., Zhang S.H., Han F.Y., Sha J.F., Zheng X. (2022). Role of silica fume on hydration and strength development of ultra-high performance concrete. Constr. Build. Mater..

[23] Luo T., Hua C., Sun Q., Tang L.Y., Yi Y., Pan X.F. (2021). Early-age hydration reaction and strength formation mechanism of solid waste silica fume modified concrete. Molecules..

[24] He Z.H., Jiang Y.Y., Shi J.Y., Qin J.H., Liu D.E., Yalcinkaya C., He Y.F. (2024). Effect of silica fume on the performance of high-early-strength UHPC prepared with magnesium ammonium phosphate cement. Case Stud. Constr. Mater..

[25] Maganti T.R., Boddepalli K.R. (2025). Synergistic enhancement of compressive strength and impact resistance in alkali-activated fiber-reinforced concrete through silica fume and hybrid fiber integration. Constr. Build. Mater..

[26] Alonso M.M., Gismera S., Blanco M.T., Lanzon M., Puertas F. (2017). Alkali-activated mortars: Workability and rheological behaviour. Constr. Build. Mater..

[27] Fang G.H., Ho W.K., Tu W.L., Zhang M.Z. (2018). Workability and mechanical properties of alkali-activated fly ash-slag concrete cured at ambient temperature. Constr. Build. Mater..

[28] Zhang G., Peng G.F., Zuo X.Y., Niu X.J., Ding H. (2023). Adding hydrated lime for improving microstructure and mechanical properties of mortar for ultra-high performance concrete. Cem. Concr. Res..

[29] (2011). Code for Design of Building Foundation.

[30] Siddique R. (2011). Utilization of silica fume in concrete: Review of hardened properties. Resour. Conserv. Recycl..

[31] (2011). Specification for Mix Proportion Design of Cement Soil.

[32] (1992). Test Method for Air Mortar and Air Milk.

[33] (2009). Standard for Test Method of Basic Properties of Construction Mortar.

[34] Li A.B., Melville B.W., Yang Y.F., Zhou S., He F., Shamseldin A.Y., Zhang G.G. (2024). The semi-empirical model for critical bed shear stress in the local scour hole downstream of a submerged structure based on turbulent velocity distribution. Appl. Ocean Res..

[35] (2019). Standard for Geotechnical Testing Method.

[36] Liu Y.W., Lu C.F., Hu X., Shi C.J. (2023). Effect of silica fume on rheology of slag-fly ash-silica fume-based geopolymer pastes with different activators. Cem. Concr. Res..

[37] Panda B., Unluer C., Tan M.J. (2018). Investigation of the rheology and strength of geopolymer mixtures for extrusion-based 3D printing. Cem. Concr. Compos..

[38] Zhang R., He H.Y., Song Y.H., Zhi X.D., Fan F. (2023). Influence of mix proportioning parameters and curing regimes on the properties of ultra-high strength alkali-activated concrete. Constr. Build. Mater..

[39] Li L., Wei Y.J., Li Z.L., Farooqi M.U. (2022). Rheological and viscoelastic characterizations of fly ash/slag/silica fume-based geopolymer. J. Clean Prod..

[40] Zhang S.L., Qi X.Q., Guo S.Y., Zhang L., Ren J. (2022). A systematic research on foamed concrete: The effects of foam content, fly ash, slag, silica fume and water-to-binder ratio. Constr. Build. Mater..

[41] Taloey N., Aikawa Y., Kubota O., Sakai E. (2018). Theoretical analysis of the hydration dependence of low heat Portland cements with or without silica fume on their packing fractions. J. Ceram. Soc. Jpn..

[42] Huang F.M., Liu J.P., Li X.C., Li C., Hu Z.L., Shen X.J., Chen B.C. (2024). Impact of silica fume on the long-term stability of cement-based materials with low water-to-binder ratio under different curing conditions. Constr. Build. Mater..

[43] Ashraf M., Iqbal M.F., Rauf M., Ashraf M.U., Ulhaq A., Muhammad H., Liu Q.F. (2022). Developing a sustainable concrete incorporating bentonite clay and silica fume: Mechanical and durability performance. J. Clean Prod..

[44] Abbas S.N., Qureshi M.I. (2025). Improved Fresh and Hardened properties of Concrete with High Density Polyethylene aggregates: Role of Silica fume, Steel Fibers, Macro synthetic fibers and Variation of water cement ratio. Mater. Chem. Phys. Sustain. Energy.

[45] Oyunbileg D., Amgalan J., Batbaatar T., Temuujin J. (2023). Evaluation of thermal and freeze-thaw resistances of the concretes with the silica fume addition at different water-cement ratio. Case Stud. Constr. Mater..

